# Difference in summer heatwave-induced damage between desert native and urban greening plants in an arid desert region

**DOI:** 10.1371/journal.pone.0299976

**Published:** 2024-12-06

**Authors:** Bo-Rui Li, Nan Sun, Ming-Shan Xu, Qi-Xing Sun, Hui-Ming Wang, Jie Zhou, Xu Luo, Guang-Hui Lv, Xiao-Dong Yang

**Affiliations:** 1 Department of Geography & Spatial Information/Center for Land and Marine Spatial Utilization and Governance Research, Ningbo University, Ningbo, China; 2 Institute of Resources and Environment Science, Xinjiang University, Urumqi, China; 3 Zhejiang Institute of Hydraulics & Estuary (Zhejiang Surveying Institute of Estuary and Coast), Hangzhou, Zhejiang, China; PLOS: Public Library of Science, UNITED STATES OF AMERICA

## Abstract

Summer heatwaves have caused a distinct mortality between urban greening and native plants. However, there are insufficient studies revealing the underlying mechanisms. We hypothesized that differentiation in hydraulic traits and their integration cause the varied heatwave-induced damages between the two plant types. To prove it, three desert native species and five urban greening species were selected as the experimental objects. Then, the number of damaged individuals caused by summer heatwaves were investigated based on the 100 individuals for each species. The hydraulic traits (including hydraulic transport, photosynthetic and leaf traits) of 3–5 mature individuals were measured for each species. The comparative analysis (independent sample t test and one-way ANOVA) and the collaborative analysis (Pearson correlation and network analysis) were used to reveal the differences in heatwave-induced damage, hydraulic traits and their integration between urban greening and native plants. Our results showed that the heatwave-induced damage to urban greening plants was larger than that to native species. Water potentials of leaf and branch in pre-dawn and midday, *P*_*50*_, leaf dry matter content, net photosynthetic rate, transpiration rate and stomatal conductance of desert native species were significantly lower than those of urban greening plants (*P* < 0.05), while twig specific hydraulic conductivity, Huber value, wood density, intrinsic water use efficiency and the specific leaf area showed opposite patterns (*P* < 0.05). Trait integration of desert native species (0.63) was much higher than greening plants (0.24). Our results indicate that artificial urban greening plants are more susceptible to drought stress caused by heatwaves than native desert species. In the context of global climate change, in order to maintain the stability and function of urban ecosystems in extreme climate, the screening of greening plants should start from the perspective of hydraulics and trait integration, and more native species with strong drought adaptability should be planted.

## 1 Introduction

Affected by the global climate change, the phenomenon of summer heatwave occurs frequently all over the world, and shows an increasing trend [1]. In 2022, for example, the temperature in the middle of June in Europe and Asia began to break through 40°C, while the highest temperature is much higher than the same previous period years (27°C) [2]. In mid-July, the highest temperatures in the Portugal, North Africa and Iran reached 45°C, 48°C and 52°C, respectively, all of which were the highest ever recorded in local history. The Met Office has issued a red alert for “extreme heat” for the first time in its history. After China issued a red alert for high temperatures on July 12, a total of 7,555 high temperatures above 40°C were recorded in 23 Chinese provinces as of August 28. 11.6 times higher than the average in the first five years of the same period (2017–2021) [3]. As a natural hazard, summer heatwaves affect public health and life safety by increasing the incidence and mortality of heat-related diseases [4]. At the same time, heatwaves increase drought stress on plants. A large number of drought-induced tree mortality due to hydraulic failure or hydraulic dysfunction alter community structure, lead to loss of biodiversity, and weaken ecosystem function and stability [5–7].

The effects of heatwaves on plants may vary from species types [8]. According to the location of plant growth and the degree of human management, plants can be divided into natural native and urban greening plants. Native plants are that grow and reproduce naturally without human factors. On the contrary, urban greening species are cultivated, maintained and managed to form urban landscape and improve urban ecological quality [9, 10]. Compared with native species, artificial greening plants can better meet the aesthetic needs of urban residents in terms of appearance and landscape improvement. At the same time, however, in order to maintain its aesthetic characters and keep it alive, urban greenery often requires the care of municipal managers or local residents [11]. As a result, artificial urban greening plants are in a favorable environment due to long-term adaptive environmental selection, and their ability to deal with natural disasters is weaker than that of native species [12]. Thus, greening plants would have a higher drought-induced mortality than native species during heatwave period.

Arid and semi-arid areas cover about one third of the land area [13]. Affected by low precipitation and high transpiration, plants in this region are generally subjected to drought stress [13–15]. Heatwaves exacerbate drought stress on plants, making them more likely to die from hydraulic failure than in humid areas [16]. This point has been proved in the research of many scholars. For example, Liu et al. found that the high temperature caused by the heatwave caused a large number of plants to die in the arid grassland, thus reducing the stability and productivity of the ecosystem [17]. The prolonged heatwave in the southwest of the United States caused considerable amount of pine deaths, especially in the arid region, resulting in continuous degradation of the ecological environment [18]. Under the influence of heatwave, a large area of shrubs died in the arid area of southern Australia, which not only damaged the ecosystem, but also made the wild herbivores lose their food source, resulting in the death of animal groups due to food shortage [19]. However, none of these studies distinguished the difference between native and green species, and even fewer examined what caused the drought-induced mortality differences between the two species during summer heatwaves.

The difference of the hydraulic traits and their integration may be the internal cause of the different mortality rates between artificial urban greening plants and desert native species during heatwave period [20–22]. It is well-known that summer heatwaves damage plants from two main ways [23, 24]. The first aspect is high temperatures irreversibly damage leaf photosystems II by inactivating the photosynthetic enzyme and destroying the photosynthetic structure [25]. The second is that high temperature damages the hydraulic system by increasing drought stress due to shortage of available water, thus affecting plant photosynthesis and carbon synthesis, resulting in drought-induced mortality due to the lack of water or organic synthesis, especially non-structural carbon [25, 26]. The above two damage processes caused by summer heatwave to plants are not independent. It is generally believed that plants can reduce leaf surface temperature through transpiration when their hydraulic system is not damaged by drought stress or when they can absorb enough water from the soil, thereby avoiding direct damage from high temperatures [23]. Therefore, the variation of hydraulic traits is used to evaluate the differential effect of summer heatwave on plants.

Hydraulic traits refer to the morphological, structural and physiological properties of plants formed during long-term evolution, such as transpiration rate, stomatal conductance, Huber value, *P*_*50*_ and branch xylem hydraulic conductivity; which are related to water absorption, transport and water vapor exchange between leaves and atmosphere [27, 28]. It has been confirmed that when plants suffer from heatwave, differences in these traits will lead to shift in plants’ ability to cope with drought stress, thus affecting mortality [5, 21, 29]. For example, species with higher *P*_*50*_ or lower water conductivity preferentially experience hydraulic imbalance, resulting in either a loss of water in the plant (hydraulic failure) or a lack of nutrients due to stomatal closure (carbon starvation), leading to death [6, 30]. Plants use stomatal regulation strategies to change stomatal conductance and water use efficiency to reduce water loss for drought resistance [31, 32]. Additionally, plants adapt to drought not through each trait alone, but through a combination of trait [3, 33]. In other words, plants use complementary relationships and trade-offs between different traits to improve their adaptability to drought [34]. This adaptation of trait combinations is called trait integration by ecologists [35, 36]. Existing studies have found that trait integration is more effective in mitigating drought stress than individual traits, and it can more accurately predict drought-induced plant deaths [31]. The higher the integrated degree of plant hydraulics, it means that plants have better adaptability to drought and are less prone to drought-induced death during the heatwave [20, 21]. However, research regarding the distinct mortality of urban greening and native desert plants caused by heatwave from the perspective of hydraulics and trait integration is relatively insufficient [37].

**Fig 1:**
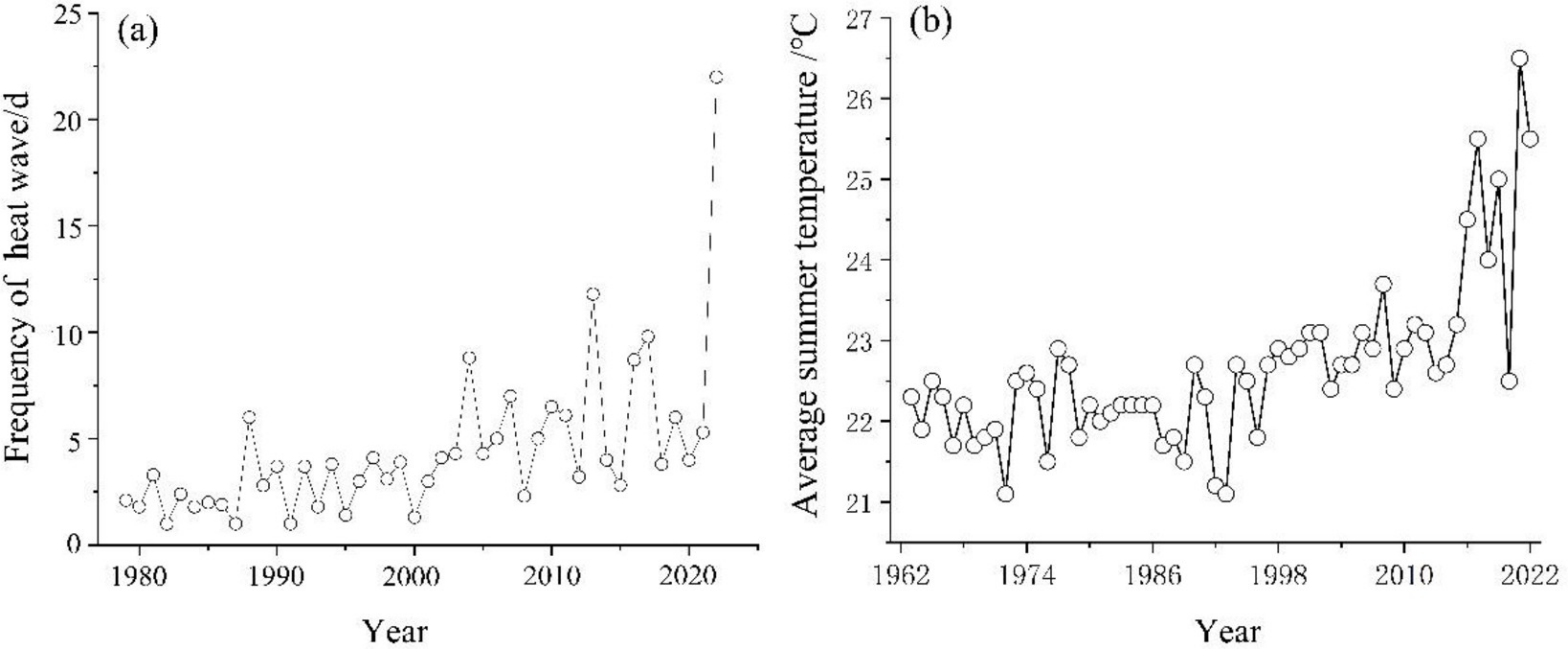
The frequency of heat wave from June to August of 1979 to 2022 (a), and the change of summer mean temperature over the past 60 years (b) in Xinjiang Uygur Autonomous Region.

China’s Xinjiang Uygur Autonomous Region is located in central Asia, and the farthest place from the ocean. The lack of rainfall and the high evaporation caused the local plants to suffer from chronic drought stress [33]. According to the Xinjiang Meteorological Center, the local average summer temperature has risen from 23.2°C to 26.4°C in recent years due to global climate change. The extreme maximum temperature increased from 32°C to 39°C (Fig 1). In 2019, 2020 and 2022, there will be consecutive summer heatwave events. Suffering more than 20 heatwaves and a maximum heat duration of 64 days, the 2022 heatwave is believed to be the longest and strongest since 1961 (Fig 1). The occurrence of summer heatwave has a serious negative impact on the local ecological environment, resulting in the death of a large number of plants.

During the summer heatwave of 2022, the Jinghe County in Xinjiang Uygur Autonomous Region, located at the western edge of the Gurbantunggut Desert, was selected as the research site to measure the differences in hydraulic traits and trait integration between three tree desert native species and five urban greening species. We hypothesize that the differentiation in hydraulic traits and their integration cause the distinct heatwave-induced damage between the urban greening and desert native plants. The answer to the following two scientific questions is used to prove it: 1. Are urban greening plants more likely to damage than desert native species during heatwaves? 2. Whether the differences in hydraulic traits and trait integration causes the distinct drought-induced damage due to heatwave between the two plants. Solving these two problems can provide important scientific basis for urban greening and ecosystem maintenance in arid areas, and also provide theoretical support for the selection of urban drought-resistant green plants.

## 2 Materials and methods

### 2.1 Study area and ethics statement

The study area is located in the Jinghe County (44°00 ’~45°10’ N, 81°46 ’~83°51’ E) at the western edge of the Gurbantunggut Desert in Xinjiang Uygur Autonomous Region of NW China. Affected by the typical continental climate, the average annual precipitation is about 100 mm and the annual average evaporation is over 1600 mm. The sunshine duration is about 2800 h, the extreme maximum temperature is 44°C, the extreme minimum temperature is -33°C, and the average annual temperature ranges from 6 to 8°C. The county is surrounded by mountains on three sides, with a trumpet-shaped valley plain in the middle, and adjacent to the Gurbantunggut Desert and the Alashan Pass. The average elevation is 189 m. The soil type was mainly gray brown desert soil with high salinization degree and *p*H value in the range of 7.52~9.29. The native plant community is arid sparse shrubbery, and the native species are mainly desert plants that are extremely drought tolerant. There are only three native desert tree species named *Populus euphratica*, *Haloxylon ammodendron* and *Tamarix chinensis*.

The selection of artificial greening plants is based on the following two points: 1. Artificial planting of green tree species; 2. It is widely distributed in the Jinghe County and is the dominant species of artificial community. According to this rule, five urban greening species were selected in this study, which were named as *Salix matsudana*, *Populus tomentosa*, *Ulmus laevis*, *Ulmus densa* and *Elaeagnus angustifolia*. In order to exclude the influence of abiotic factors such as terrain, soil types, groundwater depth and micro-climate on our analytical result, the ecotone between the Tuotuo Town and the Ebinur Desert in the Jinghe County was chosen as the experimental area; where five urban greening species and three native desert tree species all grow, and the distance between their growth locations is not more than 3 km. The Tuotuo Town is a small town in the Jinghe County. Greening plants grown in the rustic biggest park and were mainly distributed in the side close to the Tuotuo Town. They were mainly planted by local residents spontaneously or by the local greening department. In contrast, native plants grow mainly on the side near the desert. As the experimental individuals are located in a small spatial range, the similarity of various abiotic factors enables their influences on our test results to be excluded.

The experimental field owned and managed by the local government while are not privately owned or protected in any way. Thus, a specific permit for not for-profit research is not required. Our studies did not involve endangered or protected plant species in this area.

### 2.2 Investigation of plant damage caused by drought due to heat waves

As suggested from the World Meteorological Organization, summer heatwaves occur when the maximum daytime temperature exceeds 32°C, which is considered harmful to human health [38]. According to the Xinjiang Meteorological Center, the duration of the summer heat wave in the Jinghe County in 2022 ranged from June 1 to August 24 (S1 Fig). We surveyed 100 individuals of each studied species for their drought-related damage in the ecotone in the later stages of the 2022 summer heat wave period (late August). The damage of drought on plants are defined in five degrees: 1. unaffected, 2. slight; 3. moderate; 4. severe; and 5. plant death (Table 1).

**Table 1:** The degrees of plant drought-related damage due to the summer heat wave.

Grade	The extent of plant damage caused by drought	Characteristics
1	Unaffected	The plants have not changed
3	Moderate	More leaves turn yellow and lose their leaves
4	Severe	Most of the leaves fell off, some branches dried up and died
5	Plant death	The leaves fall off completely and the plant dies

### 2.3 Experimental measurement

#### 2.3.1 Selection of experimental samples

The adverse effects of summer heatwaves on plants accumulate over time. To capture the maximum damage, we measured the hydraulic traits of selected species in the later stages of summer heatwaves. 3~5 mature or lager individuals of each species in the ecotone were selected for experimental determination.

#### 2.3.2 Hydraulic traits measurement

*2*.*3*.*2*.*1*. *Pre-dawn water potential and midday water potential*. For each selected individual, 3–5 branches with complete leaves and no insect pests were randomly selected, and then were cut off. According to the method of Yang et al. [33]. The samples were pretreated and preserved. After that, the predawn and midday water potential of leaves and branches were measured by using a portable dew-point water potential meter (WP4C, Decagon Devices, Pullman, Washington, USA) and a Pressure Chamber Instrument (1505D-EXP; PMS Instrument Company, Albany, OR, USA), respectively. All potential measurements were repeated three times. The sampling time of water potential in pre-dawn and midday was 05:30–07:30 and 12:30–14:30 local time, respectively.

*2*.*3*.*2*.*2*. *Xylem hydraulic conductivity*, *P*_*50*,_ *Huber value and wood density*. Xylem hydraulic conductivity (twig specific hydraulic conductivity /*Ks* and quasi-steady-state water conductivity /*K*) was obtained by measuring the xylem embolization vulnerability curve using plant water conductivity measuring instrument (HPFM-Gen3, Dynamax Inc., Houston, USA). The sampling of branches and the subsequent experimental pretreatment were referred to Venturas et al.’s method [39]. The measurement of xylem hydraulic conductivity was suggested from Gong et al. [40]. According to the Sperry, et al. [41], a pressure chamber instrument (1505D-EXP; PMS Instrument Company, Albany, OR, USA) and high pressure flowmeters were used to determine the percentage of water conductivity loss (*PLC*; %). Then, *P*_*50*_ (the water potential at the loss of 50% water conductivity coefficient) was obtained by fitting the *PLC* curve [42]. The increment of pressure was set as 0.5 MPa, as suggested from Yang et al. [13]. After that, the wood cross-sectional area (*SA*; m^2^) and the total area of all leaves on a branch (*TLA*; m^2^) were measured using a vernier calipers and a leaf area meter (Li-3100C, Lincoln, NE, USA), respectively. The Huber value was calculated by the Eq 1. The sampling time of branches was 06:00–07:00 local time. The wood density of small branch / terminal branch (*SWD*) was measured by the drainage method. Specific method was described in detail in our previous study Yang et al. [13].


Hubervalue=SA/TLA (1)

*2*.*3*.*2*.*3*. *Leaf dry matter content and specific leaf area*. For each selected individuals, 3–5 branches with intact leaves and no insect damage were selected, and then 3–5 leaves were cut off. After all samples were brought back to the laboratory, the area (*LA*, cm^2^) and the fresh weight (*LFW*, g) of leaves was measured using the leaf area meter and an electronic analytical balance with an accuracy of < 0.000 1 g, respectively. As suggested by the method of Yamasaki and Dillenburg [43], the dry weight of leaves (calculated using leaf relative water content) (*LDW*, g) was measured. Leaf dry matter content *(LDMC*, g⋅g^−1^) and specific leaf area (*SLA*, m^2^⋅kg^−1^) was calculated by the Eqs 2–3, respectively.


LDMC=LDW/LFW (2)

SLA=LA/LDW (3)

*2*.*3*.*2*.*4*. *Transpiration rate*, *stomatal conductance*, *intrinsic water use efficiency and net photosynthetic rate*. Stomatal conductance (*Gs*, H_2_Oμmol·m^-2^·s^-1^), transpiration rate (*Tr*, H_2_Oμmol·m^-2^·s^-1^), net photosynthetic rate (*Pn*, μmolCO_2_·m^-2^·s-^1^) was measured by the portable photosynthesis measurement system (Li-6400XT, Li-COR, Lincoln, NE, USA). The specific measurement process was described in detail in the previous studies [13, 33]. To minimize the influence of microenvironment changes on photosynthetic traits, the LI-6400-2B red and blue light source of the instrument was adopted, and light intensity, temperature and CO_2_ concentration in the leaf chamber of the portable photosynthesis measurement system were set at 1600 μmol·m^-2^·s^-1^, 35°C, and 400 μmol·mol^-1^, respectively. The intrinsic water use efficiency (*WUEi*, mol·mol^-1^) was calculated by *Pn* and *Gs* (*WUEi* = *Pn* / *Gs*).

### 2.4 Data analysis

After hydraulic traits measured, the outliers were removed using box plot analysis. More specifically, if a data point is more than 1.5 times farther from the edge of the box, this point is considered an outlier and subsequently been removed. After that, the data were sorted into a new set according to plant types. Then, the independent-samples T test was used to compare the difference of hydraulic traits between desert native species (Dns) and urban greening plants (Ugp). One-way ANOVA was used to compare the difference of hydraulic traits between five artificial greening species, as well as among three native desert species because the number of independent variables or species number was greater than 2. In the statistical processes, the post hoc tests (the Tukey HSD test) were used for pin-pair comparison between different treatments for homogeneity of variance, while Tamhane’s T3 test was used for heterogeneity of variance. If there is a difference in hydraulic traits between native desert species and greening plants, it indicates that their ability to withstand heatwave is varied. Similarly, differences in hydraulic traits among different species within the same plant type indicate that these species are also varied in their ability to withstand heatwave. Pearson correlation and network analysis were used to measure the difference of trait integration of two types of plants (native desert species and greening plants) in coping with summer heatwave. The more pairs of significant correlation in Pearson correlation and the high value of integration indicated that the plants adopt complementary relationships and trade-offs between different traits to improve their adaptability to heatwave. Network analysis is commonly to explore how multiple traits are integrated [35, 36]. In trait correlation networks, traits are treated as nodes, while their connections are treated as edges. Out of all of the parameters in the network, degree represents the number of connections between focal traits and other traits, indicating the level of integration. All analysis were conducted in R statistics software (4.0 version). As suggested by Csardi and Nepusz [44], the "*igraph*" package was used to measure trait integration based on Pearson correlation coefficients.

## 3. Results

### 3.1 Difference of drought-induced damage between desert native species and artificial greening plants

**Fig 2:**
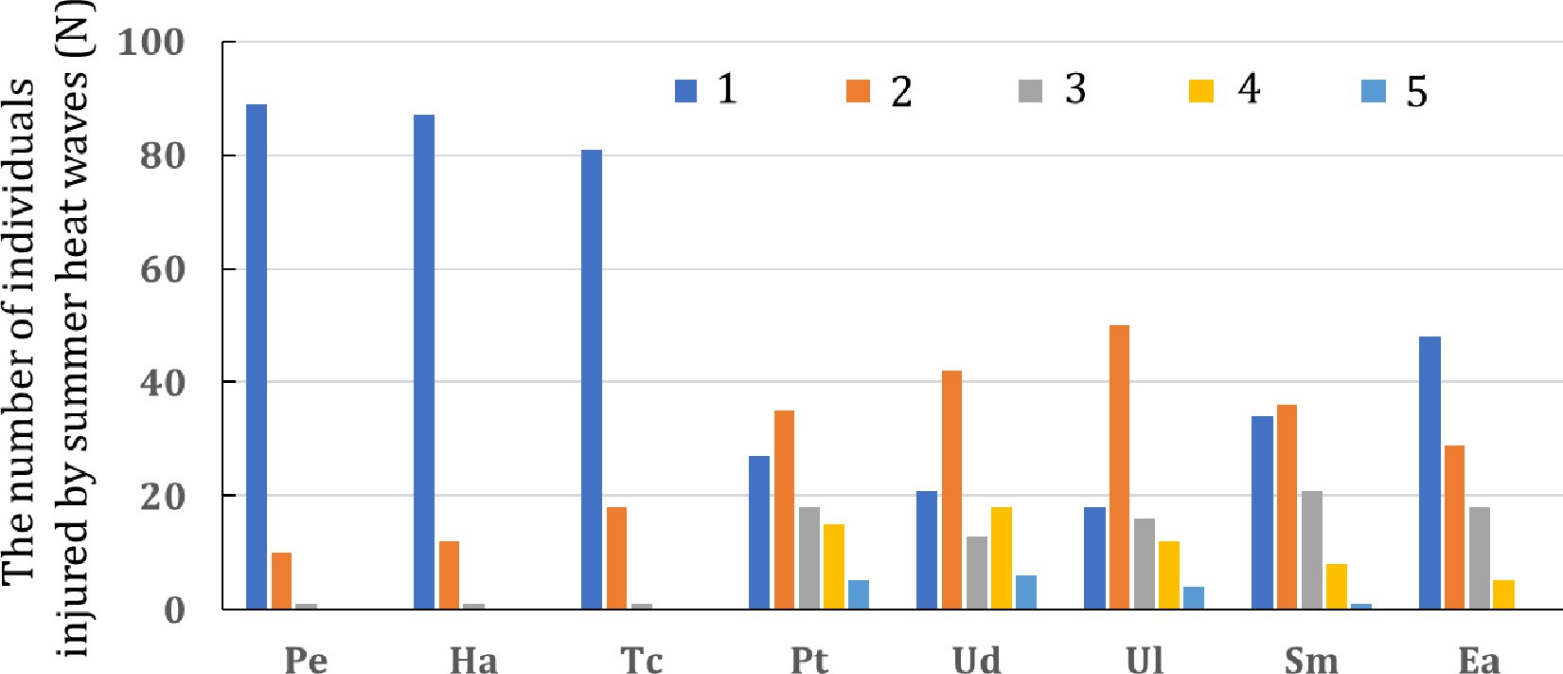
The difference in number of the drought-induced damage individuals between desert native species and artificial urban greening plants due to summer heatwave. Pe, Ha and Tc is *Populus euphratica*, *Haloxylon ammodendron* and *Tamarix chinensis*, respectively. Pt, Ud, Ul, Sm and Ea is *Populus tomentosa*, *Ulmus densa*, *Ulmus laevis*, *Salix matsudana* and *Elaeagnus angustifolia*, respectively. 1, 2, 3, 4, and 5 respectively indicate that the plants have suffered varying degrees of drought-induced damage caused by the summer heat waves, which are unaffected, slight, moderate, severe, and plant death, respectively.

There were less than 20 damaged individuals of desert native species (*P*. *euphratica*, *T*. *chinensis* and *H*. *ammodendron*) at grade 2, only 1 damaged individual at grade 3, and no damaged individuals at grade 4 and 5. More than 80% of individuals were unaffected by summer heatwaves (Fig 2). On the contrary, there were grade 4 and 5 damaged individuals in five artificial greening species. Except for no individual of *E*. *angustifolia* death caused by heatwave, *P*. *tomentosa*, *U*. *densa*, *U*. *laevis* and *S*. *matsudana* died 5, 6, 4 and 1, respectively. No damaged individuals of *P*. *tomentosa*, *U*. *densa*, *U*. *laevis*, *S*. *matsudana* and *E*. *angustifolia* only accounted for 27%, 21%, 18%, 34% and 48% of the total, respectively. The number of damaged individuals of *S*. *matsudana* and *E*. *angustifolia* above grade 2 was much lower than that of *P*. *tomentosa*, *U*. *densa* and *U*. *laevis* (Fig 2).

### 3.2 Differences in hydraulic traits between desert native species and urban greening plants

**Fig 3:**
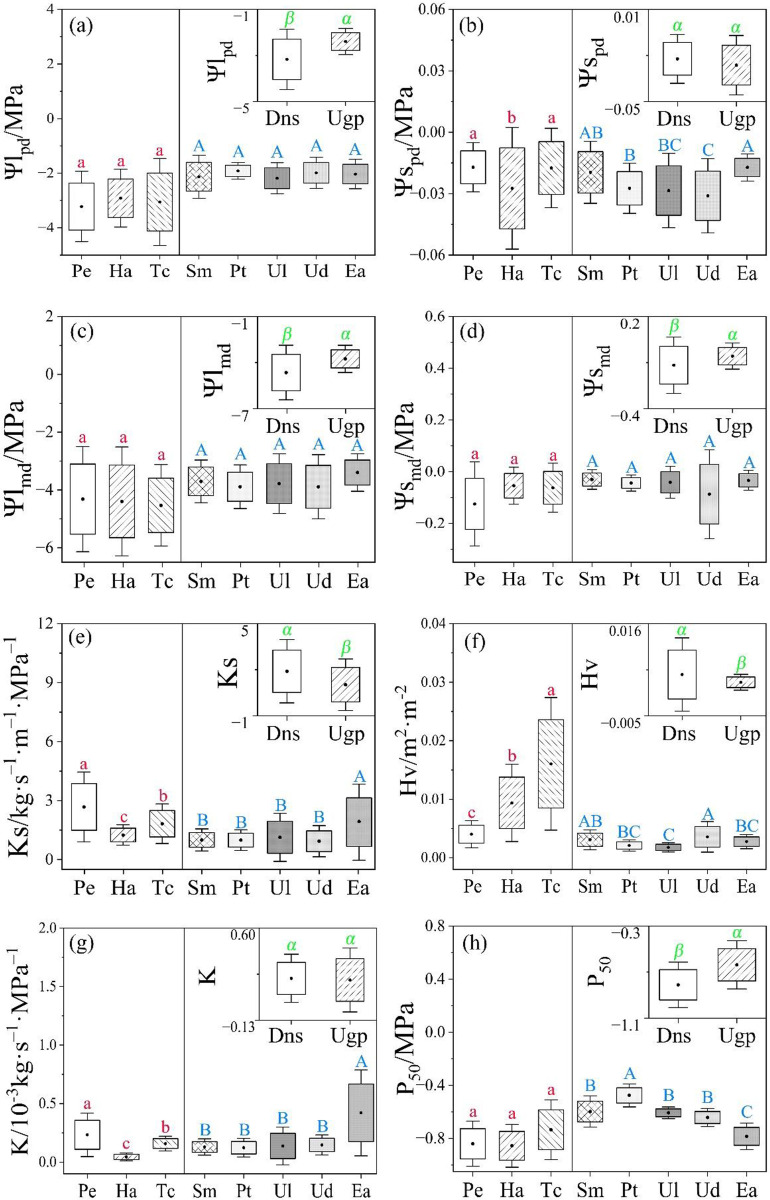
Differences in the hydraulic traits between desert native species (Dns) and urban greening plants (Ugp). Ψlpd, Ψlmd, Ψspd, Ψsmd are pre-dawn leaf water potential, midday leaf water potential; pre-dawn stem water potential and midday stem water potential, respectively. *Ks*, *K*, *Hv* and *P*_*50*_ indicate twig specific hydraulic conductivity, the quasi-steady water conductivity, Huber value and water potential at 50% loss of water conductivity, respectively. The introduction of Pe, Ha, Tc, Sm, Pt, Ul, Ud and Ea is showed in Fig 2. The small figure in the upper right corner represents the difference in hydraulics traits between desert native species and greening plants, which is tested by the independent sample T-test. The letters on either side of the solid middle line indicate the difference among species within the same type (desert native species or urban greening plants), which is measured using one-way ANOVA. The same letter indicates no significant difference between treatments, whereas different letters suggest significant differences. Green latin, bule uppercase, and red lowercase letters show the results of comparison between two plant types (greening plants and desert native species), and among species within these two plant types (red: desert native species; bule: greening plants), respectively. *P* < 0.05.

The pre-dawn leaf water potential, midday stem water potential and *P*_*50*_ of desert native plants were significantly lower than those of artificial urban greening plants, but the *Ks* and Huber value showed a completely opposite pattern, while pre-dawn stem water potential and *K* had not significant differed between two plant types (Fig 3).

For desert native plants, there were no significant differences in pre-dawn leaf water potential, midday leaf and stem water potentials, and *P*_*50*_ among the three species. However, the pre-dawn stem water potential, *Ks* and *K* of *H*. *ammodendron* were significantly lower than those of *P*. *euphratica* and *T*. *chinensis*. Among the three species, Huber value of *T*. *chinensis* was significantly higher than that of *H*. *ammodendron* and *P*. *euphratica* (Fig 3). In terms of urban greening plants, there were no significant differences in pre-dawn leaf water potential, midday leaf water potential, midday stem water potential among the five species, but there were differences in pre-dawn stem water potential, *Ks*, Huber value, *K* and *P*_*50*_. Specifically, *Ks* and *K* of *E*. *angustifolia* were significantly higher than those of the other four species, but there were no significant differences among the other three species. Huber value in *U*. *laevis*, *P*. *tomentosa* and *E*. *angustifolia* were significantly lower than that in *S*. *matsudana* and *U*. *densa*. Among five species, *P*. *tomentosa* has the highest *P*_*50*_, while the lowest was in *E*. *angustifolia* (Fig 3).

**Fig 4:**
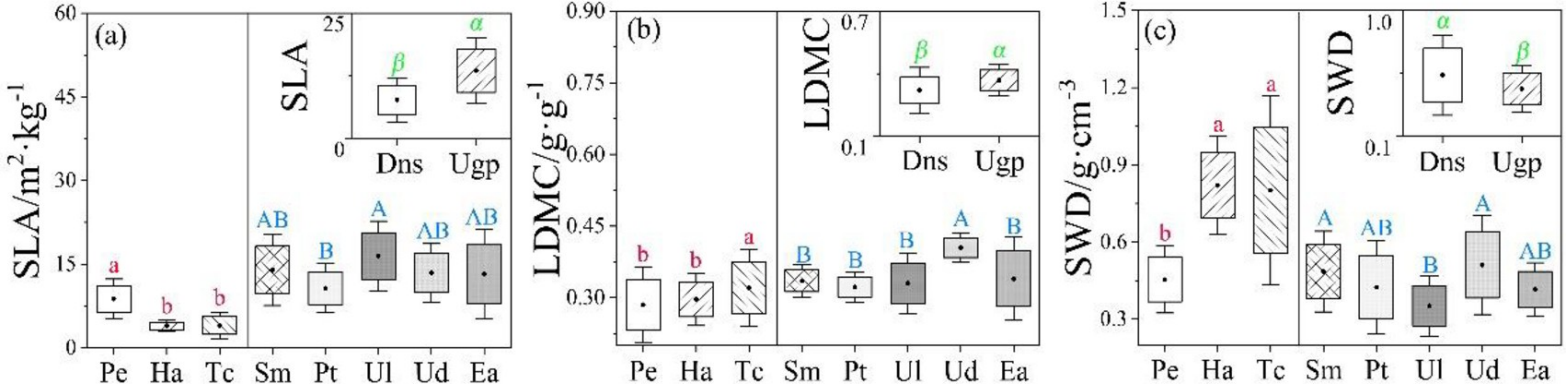
Differences in specific leaf area (*SLA*), leaf dry matter content (*LDMC*) and wood density of small branch (*SWD*) between desert native species (Dns) and urban greening plants (Ugs). The introduction of letters is show in the Fig 2 and Fig 3. *P* < 0.05.

The *SWD* of desert native species were significantly higher than those of urban greening plants, but *SLA* and *LDMC* showed an opposite pattern (Fig 4). Among three desert native species, *LDMC* and *SWD* of *T*. *chinensis* were significantly higher than those of *P*. *euphratica* and *H*. *ammodendron*, but the *SLA* showed the opposite pattern (Fig 4). For the five urban greening species, *U*. *laevis* and *P*. *tomentosa* had the highest and lowest *SLA*, respectively. *U*. *densa* had the highest *LDMC* compared with other species. *SWD* were highest in *U*. *densa* and *S*. *matsudana*, while the intermediate in *P*. *tomentosa*, *E*. *angustifolia*, while the lowest in *U*. *laevis* (Fig 4).

**Fig 5:**
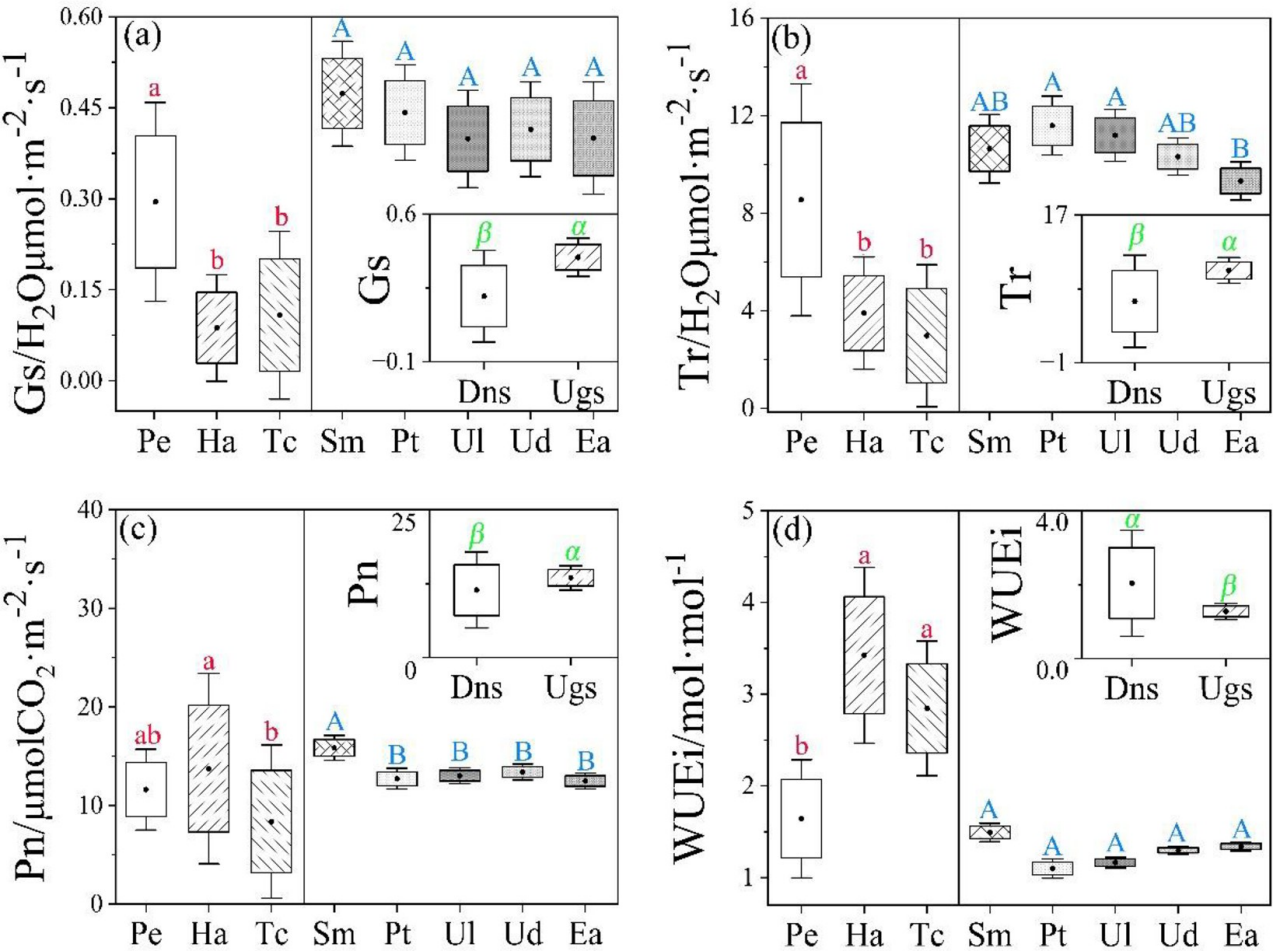
Differences in stomatal conductance (*Gs*), transpiration rate (*Tr*), net photosynthetic rate (*Pn*) and intrinsic water use efficiency (*WUEi*) between desert native species (Dns) and urban greening plants (Ugs). The introduction of letters is show in the Fig 2 and Fig 3. *P* < 0.05.

The *WUEi* of desert native species was significantly higher than that of urban greening plants, but *Gs*, *Tr* and *Pn* showed opposite trends (Fig 4). Among desert native species, *Gs* and *Tr* of *P*. *euphratica* were significantly higher than those of *H*. *ammodendron* and *T*. *chinensis*, but *WUEi* was opposite. *Pn* of *H*. *ammodendron* were higher than that of *P*. *euphratica* and *T*. *chinensis*. In terms of five urban greening species, there were no significant differences in *Gs* and *WUEi* among them, but *Tr* and *Pn* were differed significantly. The *Pn* of *S*. *matsudana* was significantly higher than that of *P*. *tomentosa*, *U*. *laevis*, *U*. *densa* and *E*. *angustifolia*. *E*. *angustifolia* had lower *Tr* compared with the other four species (Fig 5).

### 3.3 Differences in trait integration between desert native species and urban greening plants

**Fig 6:**
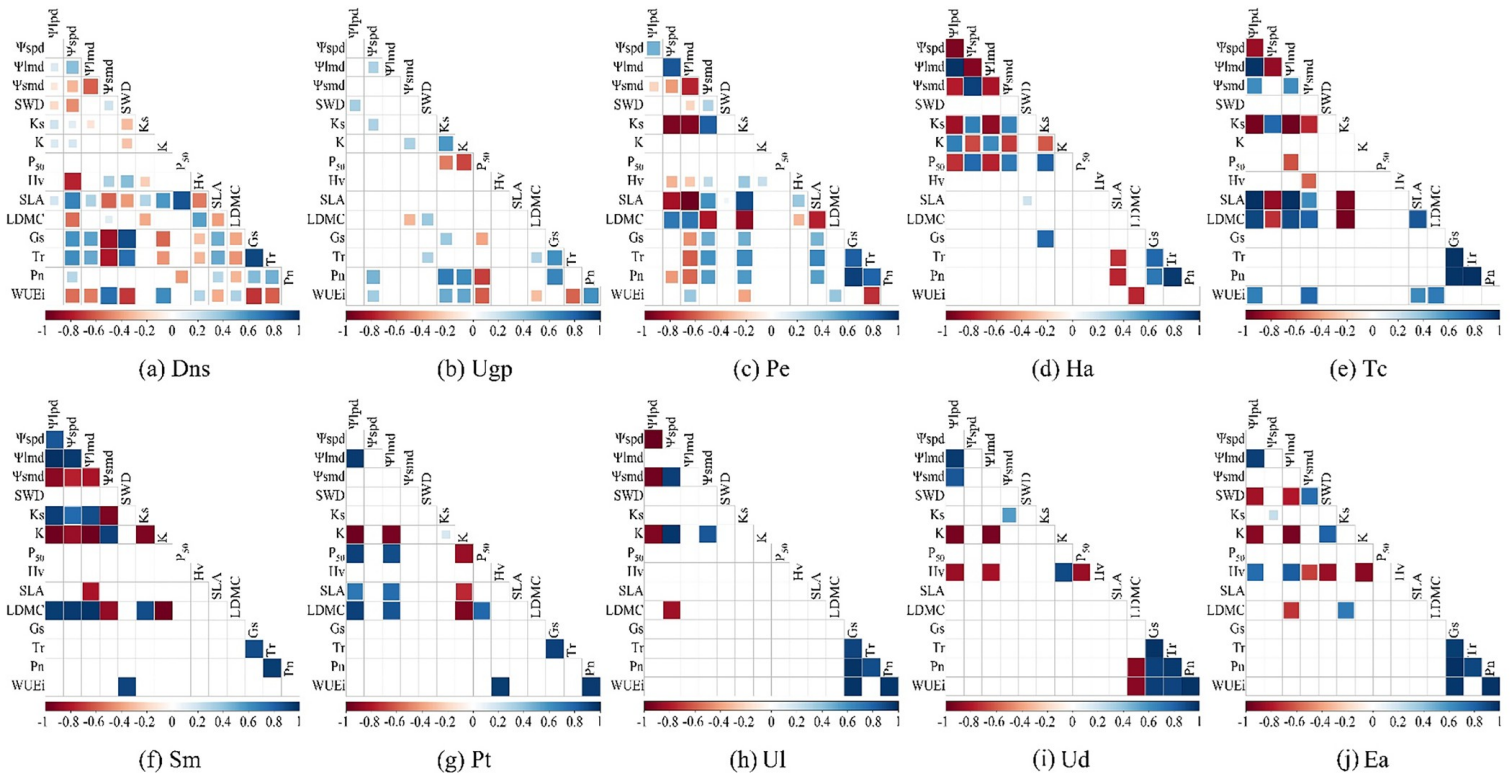
Differences in Pearson correlations among hydraulic traits between desert native species and artificial greening plants. The introduction of letters shows in the Fig 3.

The results of Pearson correlation analysis showed that the trait correlation pairs of desert native plants at 0.001, 0.01 and 0.05 confidence levels were 40, 8 and 18, respectively; which were higher than urban greening species (12, 2 and 11, respectively) (Fig 6). The correlation of functional traits of desert native plants mainly centered on pre-dawn stem water potential, midday stem water potential, *SLA*, *Gs* and *Tr* (the number of significant correlations with other traits was more than 11), while that of urban greening species mainly centered on *Ks*, *K*, *Pn* and *WUEi* (Fig 6).

For desert native species, the pairs of closely associated traits in *P*. *euphratica* were 34, 8 and 3 at the confidence levels of 0.001, 0.01 and 0.05, respectively. These values were significantly higher than those of *H*. *ammodendron* (17, 4 and 7) and *T*. *chinensis* (20, 2 and 8, respectively) (Fig 6). At the three confidence levels, the pairs of closely associated traits in the five artificial greening plants were ranked as follows: *S*. *matsudana* ≥ *E*. *angustifolia* ≥ *P*. *tomentosa* ≥ *U*. *densa* ≥ *U*. *laevis* (Fig 6).

**Fig 7:**
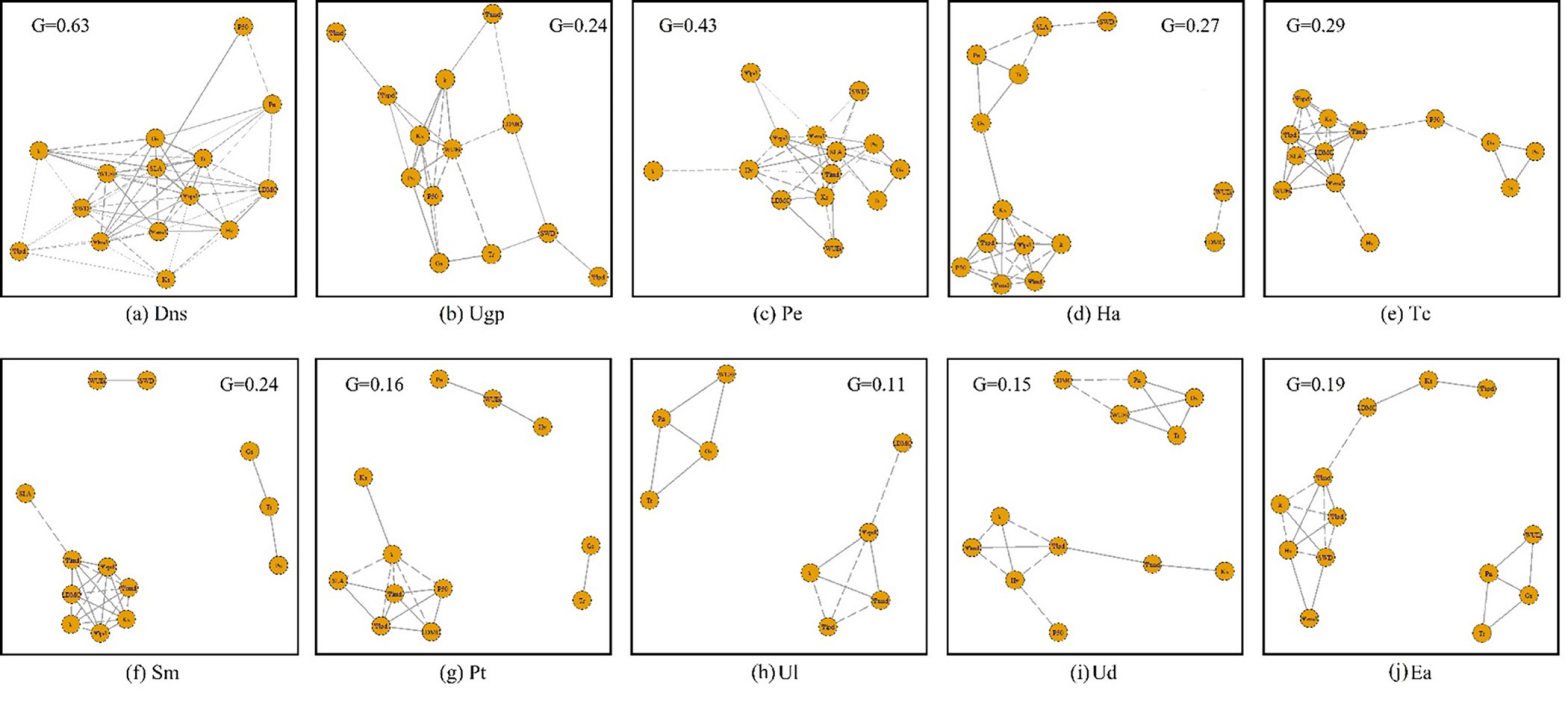
Difference in trait integration between desert native species and artificial greening plants. Positive and negative correlations among traits within each species are represented by solid and dashed lines, respectively. Line thicknesses indicate the magnitude of correlations. The introduction of letters shows in the Fig 3. *P* < 0.05.

As suggested from Flores‐Moreno et al. [35], *G* represents the network density of trait integration, and was calculated from the number of nodes *N* and boundary number *L* [*G* = 2*L*/*N*(*n*-1)]. Higher the value of *G* indicated there are higher the degree of integration among traits. It also suggested that plant have closer overall relationship among traits, and stronger adaptability to environmental stress Yang et al. [33]. Our results suggested that the degree of trait integration of desert native species (*G* = 0.63) was higher than that of artificial greening plants (*G* = 0.24) (Fig 7A and 7B). For desert native species, the key nodes of network were hydraulic and stomatal regulation-related traits (water potential, Huber value, *Gs*, *Tr*, *WUEi*), which were most closely related to other traits, and were integrated with at least 11 traits. In addition, *Ks*, *LDMC*, *Pn* and *SWD* also closely integrated with at least 4 traits (Fig 7A). In terms of artificial greening plants, *Ks*, *P*_*50*_, *Pn* and *WUEi* were the key nodes. Each of them was integrated with 7 traits (Fig 7B).

Among the three desert native species, the order of trait integration degree was *P*. *euphratica* (0.43) > *T*. *chinensis* (0.29) > *H*. *ammodendron* (0.27) (Fig 7C–7E). There were differences in the integration relationship of traits among the three desert species. All traits of *P*. *euphratica* and *T*. *chinensis* integrated into one network, whereas *H*. *ammodendron* integrated into two loose networks. One only included *WUEi* and *LDMC*, while another was consisted of hydraulic transport traits (water potential, *P*_*50*_, *Ks* and *K*) and leaf resource acquisition traits (*SLA*, *LDMC*, *Tr*, *Gs* and *Pn*). The integration of traits in *P*. *euphratica* was more compact than that in *T*. *chinensis*. The degree order of trait integration of five artificial greening plants was as follows: *S*. *matsudana* (0.24) > *E*. *angustifolia* (0.19) > *P*. *tomentosa* (0.16) > *U*. *densa* (0.15) > *U*. *laevis* (0.11) (Fig 7F–7G). There were differences in the integration relationship of traits among these five species. *S*. *matsudana*, *E*. *angustifolia*, *U*. *densa*, *U*. *laevis* and *P*. *tomentosa* all had integration relationships regarding stomatal regulatory traits to other traits, but the number of integration traits of *E*. *angustifolia*, *U*. *densa*, *U*. *laevis* and *S*. *matsudana* was significantly higher than that of *P*. *tomentosa*. In other traits, the number of integrated traits of *E*. *angustifolia* and *S*. *matsudana* was significantly higher than that of *P*. *tomentosa*, *U*. *laevis* and *U*. *densa*.

## 4. Discussion

### 4.1 Drought-induced damage of artificial greening plants was higher than desert native species

Our result indicated that the damage of summer heatwaves to artificial urban greening plants was much greater than that of desert native species. This mainly depends on two aspects: 1. compared with desert native species, urban greening plants consume more water and have less ability to resist drought stress. When selecting greening species, city management gives priority to plant appearance and other landscape characteristics (such as bright color, leaves with different characters, attractive taste, with little regard to the ability of plants to withstand environmental stress [11]. In contrast, native desert plants have evolved over time to adapt to drought [45, 46]. During summer heatwaves, the lack of drought resistance of green plants leads to increased mortality. Similar results were also found in Wu et al. ’s study [47]. They found that native plants have better drought resistance than non-native plants by increasing water transpiration, net CO_2_ assimilation. 2. The underdeveloped root system leads to increased damage of drought to greening plants during summer heat wave [48]. Greening plants grow in a privileged environment with long-term human care, causes their root system to be underdeveloped and inadequate ability to absorb water from the soil due to long-term adaptive evolution [49]. For example, Idris used modeling to confirm that the reduction in root biomass of urban woody landscape plants is an almost universal response when plants are exposed to a series of typical artificial urban environments, such as limited growing area, compacted soil, and smaller root-ball geometry [50]. This long-term environmental exposing makes urban greening plants more vulnerable to drought stress due to their inferior root biomass.

In this study, we used resident interviews to investigate differences of root system between native and greening plants. The results indicated that vertical and horizontal roots of greening plants rarely reach 3 m underground, while that of native species can reach to 5 m and over 15 m, respectively. These have been widely confirmed in several studies. The vertical roots of *H*. *ammondendron*, *P*. *euphratica* and *T*. *ramosissima* can grow below 5.5 m, 3 m and 7.57 m underground in the desert of Northwest China. Additionally their horizontal roots can grow up to 10 m, 27 m and 15 m outside the main stem [51–53]. This root advantage allows native desert plants to absorb groundwater or deeper soil water to improve their survival during summer heatwave [54]. In contrast, the underdeveloped root systems allow greening plants to grow under normal climate conditions, but after suffering summer heatwaves, higher temperatures cause soil evaporation and plant transpiration to be much higher than normal. Under this situation, the underdeveloped root system makes greening plants more difficult to absorb water from deeper parts of the soil, resulting in more damage from drought stress [40, 53]. In addition, compared with humid regions, the summer heatwave makes the soil water shortage in arid desert areas more seriously [55], further increasing the drought-induced damage on greening plants.

### 4. 2 Desert native species are more likely to survive than urban greening species due to their advantages in hydraulic traits and trait integration

Our results found that the hydraulic traits of desert native species were significantly different from those of urban greening plants. This indicates that desert native plants have greater advantages in hydraulics, such as increased water transport and water absorption capacity, and reduced water loss capacity, to counteract the damage of summer heatwaves, which makes them more likely to survive compared with urban greening plants. Water potential is an important index to measure drought resistance of plants [56–58]. In this study, the water potential of branches and leaves of desert native species at noon was lower than that of urban greening plants, indicating that desert native species could more easily absorb water from the soil than urban greening species under the influence of heatwave environment [5]. The difference between midday and pre-dawn leaf water potentials of desert species were smaller than that of greening plants, indicating that the rehydration ability of desert native species was stronger than that of urban greening plants [13]. This advantage can ensure that native desert plants have stronger drought resistance due to faster overall recovery from short-lasting drought. The benefits of rapid and high rehydration to improve plant drought resistance has been previously reported for a range of species, especially grasses and desert shrubs [59, 60].

Hydraulic structure is consisted of morphological structure of xylem vessel and water transport strategy formed by plants in order to survival under specific environmental conditions [25, 56]. In this study, the hydraulic structure (*Ks*, *K* and Huber value) of desert native species was higher than that of urban greening plants. This indicates that desert native species have higher water transport efficiency than greening plants due to better water supply per unit leaf area, and more effective xylem tissue. This makes desert native species more resistant to drought than urban plants during the summer heatwave period. Many studies have shown similar results. For example, Yang et al., found that an increase in *Ks* and *K* ensured that shrub species transported more water from the soil to the tree crown in an arid desert without xylic embolism caused by excessive transpiration forces [13]. Gong et al., found that the increase of *Ks* and *K* increased the water transport efficiency of sandy plants, thus ensuring that them grow in a more drier region in the dunes [40]. In a eucalypt forest in south-eastern Australia, the morphological adjustments of *Eucalypt obliqua*, such as the improvement of Huber value, were occurred under severe drought stress [61]. In the shrubberies in the northeastern Spain, Huber value of six dominant tree species was increased in order to resist drought along water availability gradient [29].

Leaves affect photosynthesis and water uptake from the soil through transpiration pull [33, 62]. The *Gs*, *Tr* and *Pn* of desert native species were significantly lower than those of urban greening plants, but the *WUEi* showed an opposite pattern. This indicates that desert native species need less water to synthesize organic matter. The lower water consumption of desert native species is more conducive to their adaptation to drought stress caused by summer heat wave. Similar result is also confirmed in the Wu et al. [47]. They found that plants adopt a stomatal aperture adjustment to minimize heat absorption and maximize dissipation of latent heat by increasing *Gs* while reducing *WUEi* by a PSII NPQ mechanism under severe drought stress, indicative of a water-conservation strategy in the acclimation to drought. However, this adjustment is only applicable within a certain temperature range. Because photorespiration is stimulated and photosynthesis is inhibited at high extreme temperatures. It is now suggested that heat primarily deactivates Rubisco strongly. When the heat exceeds the maximum tolerance boundary, photosynthesis stops and even leads to plant mortality because of damaged photosynthetic organs [23].

In addition, drought resistance of plants may be determined to some extent by their ability to resist the occurrence of embolism or their ability to repair after embolization [14, 15]. Wood density and *P*_*50*_ are considered to be important indexes of plant embolism resistance. Higher wood density and lower *P*_*50*_ make plant less prone to embolization [14, 57, 58, 63]. This is due to the fact that higher wood density can endure lower water potential due to lower saturated osmotic potential and osmotic potential at turgor loss point of xylem determined by higher elastic modulus, hardness of cell wall and smaller vessel diameter [56]. Many studies have found that xylem embolism accelerates diffusion and water transport function is significantly impaired in xylem vessel when the water potential is lower than *P*_*50*_ [57, 58]. Lower *P*_*50*_ ensures that the plant does not occur embolization early, thus reducing the risk of hydraulic failure. In this study, we found that the wood density of desert native species is higher than that of greening plants, while the *P*_*50*_ is opposite. These indicate that the anti-embolism ability of desert native species is stronger than that of greening plants. In the heatwave environment, desert native plants are less likely to die than urban greening species.

Our study found that trait integration of desert native species is higher than that of urban greening species. This suggests that desert native species are more drought-resistant and less damaged than greening plants in the face of heatwaves. This is because desert native plants can better coordinate the relationship among hydraulic traits (hydraulic transport, photosynthetic and leaf traits) in the face of drought, so that various ways can be adopt to minimize the negative impact of drought stress. Similar results have also been confirmed by the previous study. Liu et al. found that plants with higher levels of trait integration among were more resilient to adversity by using network analysis [20]. An experiment indicated that the integration among leaf traits of 23 desert species in high-drought stress situation was larger than that in the low-drought stress place. This coordination of plant leaf traits improved drought resistance of these species in an arid desert ecosystem [21]. In the integration of traits in desert native plants, hydraulic transport and stomatal regulation traits (water potential, *Huber value*, *Gs*, *Tr*, *WUEi*) are the key nodes of trait integration network, and are most closely related to other traits. This may indicate that desert native species mainly reduce the influence of summer heatwaves by increasing water transport and adopting stomatal regulation [7, 64]. This has been shown in numerous studies. For example, Yang et al. [33] found that plants with a higher Huber value usually have higher leaf area and photosynthetic traits. This is because the increases of leaf area, *Tr* and *Gs* increase the transpiration pull of plants and then transport more water from branches to leaves per unit time [29, 32].

In addition, desert native plants may reduce drought stress through stomatal regulation [5, 65]. Gleason et al. found that plants would close stomata to reduce the risk of hydraulic failure caused by xylem embolism due to excessive water potential difference under drought stress [62]. However, another way of the stomatal regulation is also appeared in desert native species. For example, Yang et al. found that when suffering from drought stress, the stomata of *P*. *euphratica* would not be closed, but kept open [13]. The “isohydric regulation” strategy is adopted to absorb and transport more water by increasing transpiration pull to reduce the drought stress [66, 67]. On the contrary, the traits related to stomatal regulation (*Gs*, *Tr*, *WUEi*) were most closely related to other traits in the trait integration of greening plants. This indicates that urban greening plants have a single strategy to deal with drought stress when they suffer from summer heat wave. In the short term, they can only reduce water evaporation by closing stomata and hydraulic failure caused by increased probability of embolism. After stomatal closure, the rapid loss of non-structural carbon in the plant body makes the plant constantly damaged and even die due to “carbon starvation” [6, 30]. In addition, when stomata of leaves are closed, greening plants cannot transport water to leaves, and thus cannot reduce the irreversible burn of leaves caused by high temperature through water transpiration. In this scenario, as suggested by the studies, high temperatures may inactivate the photosynthase and destroy the photosynthetic II system, leading to plant death [23, 24].

Notably, the correlation between *P*_*50*_ and other traits was opposite between desert native species and greening plants. *P*_*50*_ in desert native plants is rarely associated with other traits, but it is a key node in the integration of traits in urban greening plants. The reason may be that the relationship between xylem embolization vulnerability and short-term drought resistance of plants is not obvious. This is because, in arid environments, desert native plants are subjected to drought stress for a long time, causing xylem ducts to become permanently blocked [13, 45]. This makes drought stress more likely to cause greater xylem damage and embolism in plants in drought-prone environments such as deserts [22]. Such phenomenon is defined as “cavitation fatigue” [22, 68]. For example, Alder et al. found that the water conductivity loss of *P*. *angustifolia* was 9% without embolization at −0.5MPa water potential, whereas upped to 50% after embolization [69]. When the water conductivity loss reached 90%, the water potential was about −3.5 MPa, indicating that the plants may have died, whereas the desert species were still alive while their leaf midday water potential was close to −4 MPa. Hence, lower correlation between xylem embolism resistance and other traits are more likely to occur in drier environments [20, 21, 70]. However, in terms of artificial greening plants, *P*_*50*_ were closely related to *Ks*, *K*, and photosynthetic traits. This is because greening plants are artificially managed, especially watered regularly, and do not have the status of drought stress. During summer heatwaves, short-term increases in water consumption caused the plants to switch from not being under drought stress to being under drought stress. Water deficit widens the water potential gap between soil and leaves, and causes the negative pressure of plant xylem to increase continuously, which further leads to embolism occurrence and hydraulic failure [40, 57, 58]. In this case, water conductivity and photosynthetic traits of plants will be affected [6, 7].

Among the three desert species, trait integration of *P*. *euphratica* was higher than that of *T*. *chinensis* and *H*. *ammodendron*. This indicates that the survival strategies of *P*. *euphratica* to cope with drought stress are more prominent. Compared with *H*. *ammodendron* and *T*. *chinensis*, *P*. *euphratica* adopted more combinations and adaptation strategies to eliminate the effects of drought stress. This is because the individual of *P*. *euphratica* is larger and taller than the other two native desert species. The greater water consumption and the longer length of water transport from the soil to the crown makes it need to overcome more xylem friction and gravity relative with other two species. In order to transport more water via xylem vessels, *P*. *euphratica* is subjected to a greater leaf-to-atmosphere vapor pressure deficit (leaf-to-air VPD) and xylem tension, which requires more trait integration to offset the risk of hydraulic failure [7, 64, 71]. Similar results have appeared in Yang et al’s study [13]. They found that small tree species use more trait integration to overcome more drought stress caused by insufficient water availability than shrub species in an identical extreme drought habitat. Among the five greening plants, trait integration of *S*. *matsudana* and *E*. *angustifolia* was higher than that of *P*. *tomentosa*, *U*. *laevis* and *U*. *densa*, suggesting that the *S*. *matsudana* and *E*. *angustifolia* are less damaged in response to a summer heatwave. This can be evidenced by the number of damaged individuals surveyed during the late August. The number of damaged individuals above grade 4 of *E*. *angustifolia* and *S*. *matsudana* was much lower than that of *P*. *tomentosa*, *U*. *laevis* and *U*. *densa*. The reason for this phenomenon may come from the difference of hydraulic traits [29, 70]. For example, *Ks* of *E*. *angustifolia* is higher than that of *S*. *matsudana*, *P*. *tomentosa*, *U*. *laevis* and *U*. *densa*. This shows that the efficiency of water transport capacity of *E*. *angustifolia* from its branches to leaves is higher than that of other species. The *P*_*50*_ of *E*. *angustifolia* is lower than that of *S*. *matsudana*, *P*. *tomentosa*, *U*. *laevis* and *U*. *densa*, indicating that the anti-embolism ability of *E*. *angustifolia* is stronger than that of the other four species. Under the influence of heatwave, *E*. *angustifolia* is more resistant to the drought stress and less prone to hydraulic failure or hydraulic dysfunction caused by embolization. In addition, due to the low *Pn*, *Gs* and *Tr*, the water vapor exchange ability between the leaves and the atmosphere is also low, which is also conducive to the reduction of water loss during the summer heat wave. On the contrary, *P*. *tomentosa*, *U*. *laevis* and *U*. *densa* were more affected by summer heatwave. In addition to the high-water vapor exchange between their leaves and the atmosphere, their lower water conductivity and higher *P*_*50*_ makes their mortality rate higher compared with *E*. *angustifolia*. However, in our study region, *P*. *tomentosa*, *U*. *laevis*, and *U*. *densa* were the species with the highest planting densities. *P*. *tomentosa*, *U*. *laevis* and *U*. *densa* are planted in large numbers by the landscaping department because they are more attractive in appearance. This mismanagement led to drought-induced deaths of large numbers of greenery during summer heatwaves.

## 5. Conclusion and suggestion

Our results show that the drought-induced damages of the desert native species are far lower than the urban greening plants during the extreme summer heatwave period in 2022. As we know, the garden department mainly considered the appearance and landscape characteristics (such as flower color, smell and tree crown shape) to screen artificial greening plants, while rarely involved the ability to resist extreme environmental stress. When suffering summer heatwave, the disadvantage of underdeveloped root system and less able to absorb water from the soil makes them more vulnerable to drought stress. To reduce the impact of summer heatwave on artificial urban greening plants, it is suggested that urban landscaping managers should simulate drought from time to time after planting plants, so as to induce plant root development and improve the ability to absorb water from soil.

The mortality and the number of damaged individuals of urban greening plants are higher than those of desert native species during summer heatwave, which is also related to their weak hydraulic traits and trait integration. Since greening plants have not been subjected to drought stress, they have disadvantages in hydraulic traits, such as higher *P*_*50*_ and photosynthetic traits, and lower xylem water conductivity, making them more prone to hydraulic failure caused by embolism under drought stress. Moreover, unlike desert native species, artificial urban greening plants have the simple adaptation strategy to resist summer heatwave. They only use stomatal regulation to reduce evaporative water loss. However, due to the closure of stomata and the constant consumption of non-structural carbon in the body, they are prone to individual injury or even death due to carbon starvation. These suggests that in cities in arid desert areas, the selection of urban greening species should give priority to local native species to increase the survival rate during summer heatwaves period.

The 27th Conference of the Parties (COP27) to the United Nations Framework Convention on Climate Change in Sharm el-Sheikh of Egypt in November 2022, said that the Paris agreement’s target of 1.5°C by 2050 was now out of reach because countries had failed to take serious action to cut greenhouse gas emissions. Although we know that the summer heatwave in 2022 is the hottest and most severe on record, we don’t know if it is the warmest on record or if it is the coolest in coming decades due to the increasing carbon emission. In order to ensure the survival of urban greening plants and the health of urban ecosystems, we should pay attention to the influence of summer heatwave on greening plants, and select species with strong water transport capacity, low photosynthetic water consumption and high trait integration as greening plants from the perspective of hydraulics. Urban greening in arid areas should plant more native desert species adapted to the drought environment.

## Supporting information

S1 Fig The maximum daily temperature of the Jinghe County from June to August in summer 2022. (DOCX)

S1 Table The statistical result of the independent-samples T test for the difference in the hydraulic traits between desert native species and urban greening plants. (DOCX)

S2 Table The statistical result of the one-way ANOVA for the difference in the hydraulic traits between three desert native species. (DOCX)

S3 Table The statistical result of the one-way ANOVA for the difference in the hydraulic traits among five urban greening species. (DOCX)
