# Supplementary material for: Difference in summer heatwave-induced damage between desert native and urban greening plants in an arid desert region
Source: PLoS One. 2024 Dec 6;19(12):e0299976. doi: 10.1371/journal.pone.0299976 (PMC11623472; doi:10.1371/journal.pone.0299976)
Supplement: S1 Fig — (DOCX) [file pone.0299976.s001.docx]

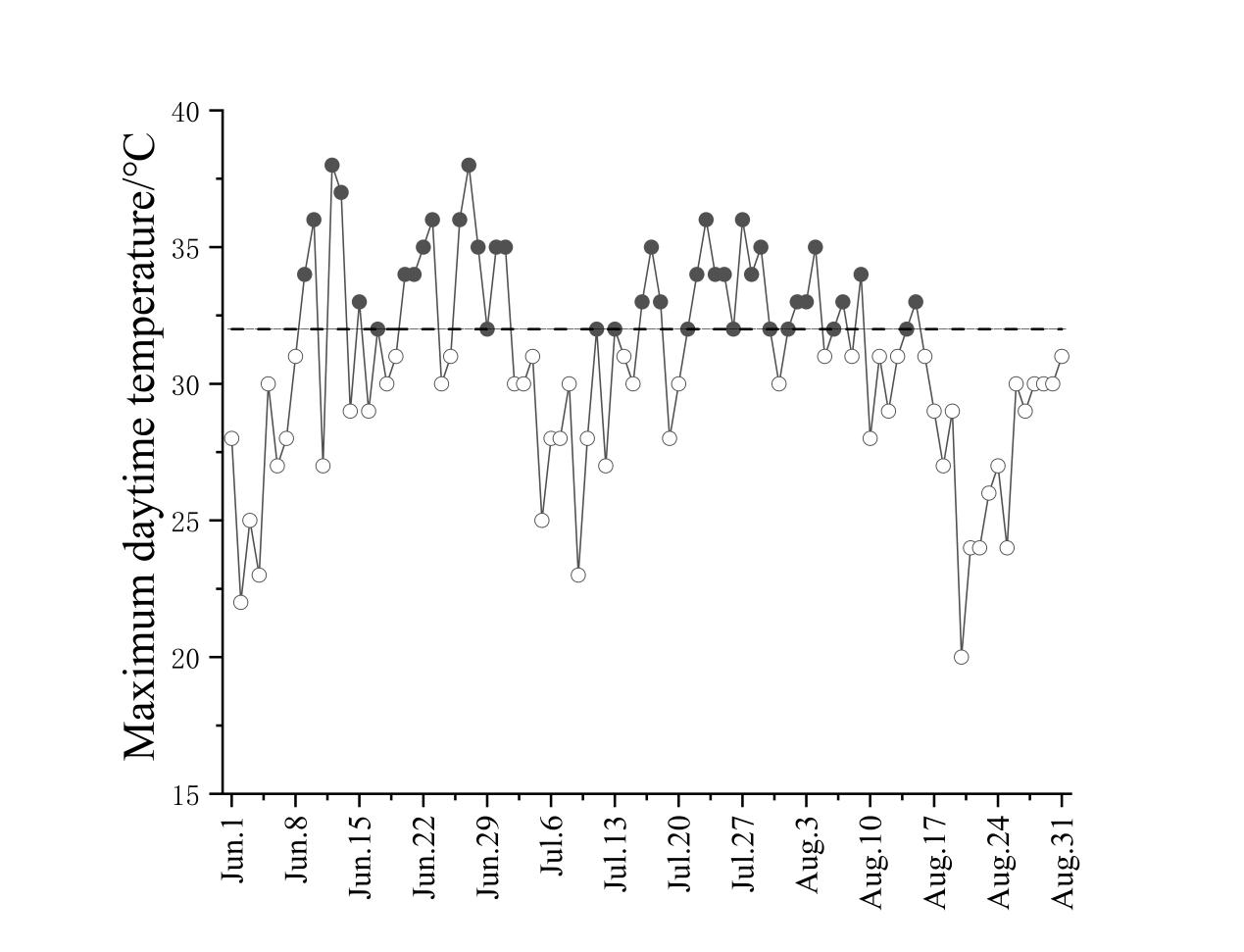


**Fig.S1.** The maximum daily temperature of the Jinghe County from June to August in summer 2022. Summer heat waves occur when the maximum daytime temperature exceeds 32℃.
