# Supplementary material for: Difference in summer heatwave-induced damage between desert native and urban greening plants in an arid desert region
Source: PLoS One. 2024 Dec 6;19(12):e0299976. doi: 10.1371/journal.pone.0299976 (PMC11623472; doi:10.1371/journal.pone.0299976)
Supplement: S1 Table — (DOCX) [file pone.0299976.s002.docx]

# Table S1. The statistical result of the independent-samples T test for the difference in the hydraulic traits between desert native species and ariticial greening plants

| **Functional traits** | **Abbreviation** | **Unit** | **t** | ***p*-values** |
| --- | --- | --- | --- | --- |
| Pre-dawn leaf water potential | Ψ_lpd_ | MPa | -10.43 | 0.00 |
| Pre-dawn stem water potential | Ψ_spd_ | MPa | 4.33 | 0.00 |
| Midday leaf water potential | Ψ_lmd_ | MPa | -2.05 | 0.04 |
| Midday stem water potential | Ψ_smd_ | MPa | -5.49 | 0.00 |
| Twig specific hydraulic conductivity | Ks | kg·s^–1^·m^–1^·MPa^–1^ | 4.65 | 0.00 |
| Huber value | Hv | m^2^·m^-2^ | 4.34 | 0.00 |
| Wood density of small branch | SWD | g·cm^-3^ | 4.14 | 0.00 |
| Quasi-steady-state water conductivity | K | 10^-3^kg·s^–1^·MPa^–1^ | 0.58 | 0.56 |
| Water potential at 50% loss of water conductivity | P_50_ | MPa | -7.55 | 0.00 |
| Specific leaf area | SLA | m^2^·kg^-1^ | -8.617 | 0.00 |
| Leaf dry matter content | LDMC | g·g^-1^ | -4.01 | 0.00 |
| Stomatal conductance | Gs | H_2_Oμmol·m^-2^·s^-1^ | -6.83 | 0.00 |
| Transpiration rate | Tr | H_2_Oμmol·m^-2^·s^-1^ | -6.91 | 0.00 |
| Net photosynthetic rate | Pn | μmolCO_2_·m^-2^·s^-1^ | -3.26 | 0.00 |
| Intrinsic water use efficiency | WUEi | mol·mol^-1^ | 5.60 | 0.00 |

Note: ***p* < 0.01, **p* < 0.05.
