# Supplementary material for: Difference in summer heatwave-induced damage between desert native and urban greening plants in an arid desert region
Source: PLoS One. 2024 Dec 6;19(12):e0299976. doi: 10.1371/journal.pone.0299976 (PMC11623472; doi:10.1371/journal.pone.0299976)
Supplement: S3 Table — (DOCX) [file pone.0299976.s004.docx]

# Table S3. The statistical result of the One-Way ANOVA for the difference in the hydraulic traits among five urban greening species

| **Functional traits** | **Abbreviation** | **Unit** | ***F*** | ***p*-values** |
| --- | --- | --- | --- | --- |
| Pre-dawn leaf water potential | Ψ_lpd_ | MPa | 0.740 | 0.569 |
| Pre-dawn stem water potential | Ψ_spd_ | MPa | 8.827 | 0.00 |
| Midday leaf water potential | Ψ_lmd_ | MPa | 0.941 | 0.452 |
| Midday stem water potential | Ψ_smd_ | MPa | 1.394 | 0.254 |
| Twig specific hydraulic conductivity | Ks | kg·s^–1^·m^–1^·MPa^–1^ | 8.30 | 0.00 |
| Huber value | Hv | m^2^·m^-2^ | 4.169 | 0.006 |
| Wood density of small branch | SWD | g·cm^-3^ | 3.309 | 0.020 |
| Quasi-steady-state water conductivity | K | 10^-3^kg·s^–1^·MPa^–1^ | 9.287 | 0.00 |
| Water potential at 50% loss of water conductivity | P_50_ | MPa | 53.362 | 0.00 |
| Specific leaf area | SLA | m^2^·kg^-1^ | 2.237 | 0.082 |
| Leaf dry mass per area | LMA | kg·m^-2^ | 1.952 | 0.121 |
| Leaf dry matter content | LDMC | g·g^-1^ | 7.471 | 0.00 |
| Stomatal conductance | Gs | H_2_Oμmol·m^-2^·s^-1^ | 2.951 | 0.032 |
| Transpiration rate | Tr | H_2_Oμmol·m^-2^·s^-1^ | 13.385 | 0.00** |
| Net photosynthetic rate | Pn | μmolCO_2_·m^-2^·s^-1^ | 40.092 | 0.00 |
| Intrinsic water use efficiency | WUEi | mol·mol^-1^ | 82.667 | 0.00 |
